# *Agrimonia pilosa* Extract Alleviates CDAHFD-Induced Non-Alcoholic Steatohepatitis and Fibrosis in Mice

**DOI:** 10.3390/nu18010042

**Published:** 2025-12-22

**Authors:** Min-Jeong Jo, Sun Jin Hwang, Myung-Gi Seo, Jun-Ho Lee, Jae Woo Lee, Yoon Hee Kim, Yongduk Kim, Sang-Joon Park

**Affiliations:** 1 Department of Histology, College of Veterinary Medicine, Kyungpook National University, Daegu-si 41566, North Gyeongsang-do, Republic of Korea; cmjdgt@naver.com (M.-J.J.); lifelove@korea.kr (S.J.H.); newmoon93@daum.net (M.-G.S.); jun25253337@gmail.com (J.-H.L.); 2 BTC Corporation, Gwacheon Creation Tower, 46, Gwacheon-daero 7-gil, Gwacheon-si 13840, Gyeonggi-do, Republic of Korea; jwlee@btcbio.com (J.W.L.); kyh@btcbio.com (Y.H.K.); dogulas.kim@btcbio.com (Y.K.)

**Keywords:** non-alcoholic steatohepatitis, *Agrimonia pilosa*, lipid metabolism, inflammation, oxidative stress, AMPK/SIRT1 signaling pathway

## Abstract

Background: Non-alcoholic steatohepatitis (NASH) lacks approved pharmacotherapies despite affecting approximately 25% of the global population. *Agrimonia pilosa*, a traditional herb with anti-inflammatory and antioxidant properties, remains unexplored for NASH treatment. Objective: This study investigated the hepatoprotective effects and mechanisms of *Agrimonia pilosa* extract (APE) in NASH models. Methods: HepG2 cells were treated with free fatty acids (0.125 mM) and APE (+12.5–50 μg/mL). C57BL/6J mice received a choline-deficient, L-amino acid-defined, high-fat diet (CDAHFD) for 12 weeks with APE (25–100 mg/kg/day), silymarin (100 mg/kg/day), or luteolin (20 mg/kg/day). Lipid accumulation, liver enzymes, histopathology, and molecular markers were assessed. Results: APE dose-dependently reduced lipid accumulation in FFA-treated cells, suppressed lipogenic factors (*SREBF1*, *CEBPA*, and *PPARG*), and upregulated fatty acid oxidation enzymes (*CPT1A* and *PPARA*) via AMPK/SIRT1 activation. In NASH mice, APE (100 mg/kg) significantly decreased serum ALT (160.0 ± 49.1 vs. 311.2 ± 66.7 U/L) and AST (96.0 ± 18.7 vs. 219.0 ± 55.7 U/L, *p* < 0.001), reduced hepatic macrophage infiltration by 68%, and substantially attenuated inflammatory markers (*Ccl2*, *Tnf*, and *IL6*), oxidative stress indicators (*NRF2*, *HMOX1*, and *CYBB*), and fibrogenic markers (*ACTA2*, *COL1A1*, and *TGFB1*) by 83–85% (*p* < 0.001). Collagen deposition decreased from 5.63 ± 0.39% to 1.54 ± 0.03% (*p* < 0.001). Conclusions: APE exerts potent hepatoprotective effects through multi-targeted modulation of lipid metabolism, inflammation, oxidative stress, and fibrosis via AMPK/SIRT1 pathway activation, supporting its potential as a natural therapeutic intervention for NASH.

## 1. Introduction

Non-alcoholic fatty liver disease (NAFLD) represents a metabolic disorder affecting approximately 25% of the global population, making it a significant public health challenge [1,2]. NAFLD encompasses a disease spectrum ranging from simple hepatic steatosis to non-alcoholic steatohepatitis (NASH), characterized by hepatic inflammation and fibrosis in the absence of excessive alcohol consumption. Without therapeutic intervention, NASH may progress to life-threatening conditions, including compensated and decompensated cirrhosis and hepatocellular carcinoma [1,3]. Additionally, NASH demonstrates strong associations with metabolic complications such as obesity, dyslipidemia, insulin resistance, and chronic kidney disease [4,5,6,7,8,9]. Despite the growing clinical need, no pharmaceutical agents have received specific approval for NAFLD treatment.

Various therapeutic strategies, encompassing both synthetic drugs and natural compounds, have undergone evaluation for NAFLD management. Medications, including pioglitazone, vitamin E, and obeticholic acid, have demonstrated promising outcomes in clinical investigations [10,11,12,13]. Pioglitazone, a thiazolidinedione class insulin sensitizer, improves hepatic steatosis, lipid metabolism, and insulin sensitivity in NAFLD patients. Vitamin E, functioning as a potent antioxidant, enhances liver biochemistry and histology in individuals with NASH. Obeticholic acid, a farnesoid X receptor agonist, reduces hepatic fibrosis in NASH patients. However, these therapeutic options face limitations including potential adverse effects and insufficient long-term safety data.

Given that inflammation and oxidative stress constitute primary drivers of NASH progression and recurrence [14,15,16], therapeutic interventions targeting these pathways with minimal adverse effects are urgently needed. Excessive hepatic fat accumulation triggers inflammatory responses through Kupffer cell activation, resulting in proinflammatory cytokine release and immune system activation. This inflammatory cascade further amplifies reactive oxygen species (ROS) production, establishing a self-perpetuating cycle of inflammation and oxidative damage that exacerbates NASH pathology. Consequently, natural compounds possessing anti-inflammatory and antioxidant properties hold substantial promise for NASH prevention and treatment.

*Agrimonia pilosa* (also termed hairy agrimony or Chinese agrimony) is a perennial herb indigenous to Asia with centuries of traditional medicinal use for conditions including fever, diarrhea, dysentery, hepatobiliary disorders, and inflammatory diseases. The plant contains diverse bioactive constituents, including triterpenoids, phenolic acids, tannins, and flavonoids, recognized for their antioxidant and anti-inflammatory characteristics [17,18]. Recent investigations have focused on *A. pilosa*’s potential as a natural therapeutic agent, demonstrating anti-inflammatory effects in lipopolysaccharide-stimulated macrophages and capacity to reduce proinflammatory cytokine production in animal inflammation models [19,20,21]. Additionally, *A. pilosa* extract (APE) has shown efficacy in ameliorating hyperglycemia and hepatic steatosis in a postmenopausal metabolic syndrome mouse model. Although some natural products have been explored as potential NASH therapeutics, the number remains limited, and few have been validated in both in vitro and in vivo NASH models. Nevertheless, *A. pilosa*’s therapeutic potential for NASH remains inadequately investigated, particularly in clinically relevant experimental models that recapitulate the key pathological features of human NASH.

To the best of our knowledge, this is the first report demonstrating the protective efficacy of A. pilosa extract in a CDAHFD-induced NASH model and HepG2 hepatocyte model, supported by mechanistic evidence involving metabolic and inflammatory regulation.

## 2. Materials and Methods

### 2.1. Preparation of APE

The APE used in this study was provided by BTC Co., Ltd. (Gwacheon, Republic of Korea; Batch No.: AEES200721-01). The extract was produced under GMP-equivalent quality standards, and a certificate of analysis (CoA) was issued confirming chemical identity, purity, luteolin-7-*O*-glucuronide content, and microbiological safety parameters. Dried *A. pilosa* leaves were extracted with 50% ethanol (Ducksan, Ansan, Republic of Korea) at 80 °C for 2 h in duplicate. First and second extracts were collected separately following filtration. The supernatants from both extractions were thoroughly combined, concentrated via vacuum evaporation, mixed with γ-cyclodextrin and dehydrated to yield APE. The material was stored at 4 °C in a sealed container until use.

### 2.2. High-Performance Liquid Chromatography (HPLC) Analysis

**Figure 1:**
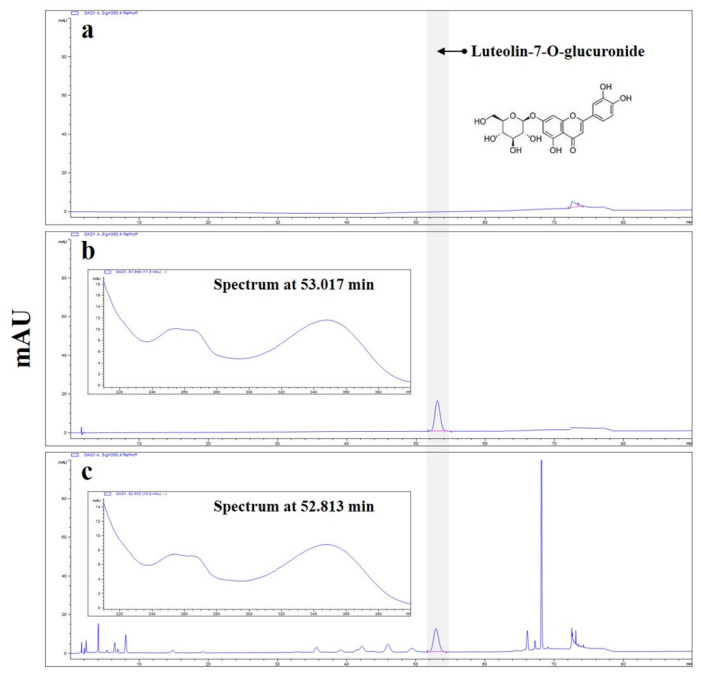
High-performance liquid chromatography with diode array detection (HPLC-DAD) analysis of luteolin-7-*O*-glucuronide *in A. pilosa* Extract (APE). The blue lines represent the HPLC-DAD UV chromatograms and corresponding UV spectra. (**a**) Blank chromatogram showing the mobile phase without sample injection. (**b**) Chromatogram of the luteolin-7-*O*-glucuronide standard. (**c**) Representative chromatogram of APE, recorded at 350 nm. The highlighted peak (red marker) corresponds to luteolin-7-*O*-glucuronide, and the chromatogram was baseline-corrected to display a single, distinct peak.

The concentration of luteolin-7-*O*-glucuronide in APE was quantified using an Agilent Infinity 1260 series HPLC system equipped with a diode array detector (Agilent Technologies, Palo Alto, CA, USA). Chromatographic separation was performed at 30 °C using an XSelect HSS C18 column (4.6 × 250 mm, 5 μm; Waters, Milford, MA, USA). The mobile phase consisted of 0.1% phosphoric acid (≥85%, Sigma-Aldrich, St. Louis, MO, USA) in distilled water (solvent A) and acetonitrile (HPLC grade; Merck, Darmstadt, Germany) as solvent B. Gradient elution was performed as follows: 0–20 min, 10% B; 20–70 min, 10–30% B; 70–75 min, 30–80% B; 75–76 min, 80–10% B; 76–90 min, 10% B. The flow rate was 1.6 mL/min, and the injection volume was 5 μL. Representative HPLC chromatograms recorded at 350 nm are shown in Figure 1. APE was standardized to contain at least 5.5 mg/g (0.55% *w*/*w*) luteolin-7-*O*-glucuronide. Quantification was carried out using a validated HPLC method. Standard solutions of luteolin-7-*O*-glucuronide (purity ≥98%, Cayman Chemical, Ann Arbor, MI, USA) at five concentrations (4–180 ppm) were analyzed in triplicate. Peak areas were plotted against concentrations, and linear regression yielded a correlation coefficient (R^2^) of 0.999, confirming excellent linearity. The limit of quantitation (LOQ), calculated as three times the baseline noise of standard injections, was 0.44 mg/g, approximately 12.5 times lower than the luteolin-7-*O*-glucuronide content in APE.

### 2.3. Cell Culture

The human hepatocellular carcinoma cell line HepG2 was obtained from the American Type Culture Collection (Rockville, MD, USA). Cells were maintained in Eagle’s Minimum Essential Medium (EMEM; Hyclone, South Logan, UT, USA) supplemented with 10% fetal bovine serum (FBS), 100 U/mL penicillin, and 100 U/mL streptomycin (Gibco, MA, USA) in a humidified incubator containing 5% CO_2_ at 37 °C. All experiments were conducted at 80% cellular confluence.

### 2.4. Preparation of Fatty Acid Stock Solution

FFA-BSA conjugate was prepared by conjugating palmitic acid (PA) and oleic acid (OA) with fatty acid-free bovine serum albumin (BSA) according to the following protocol. PA (0.0641 g) and OA (79 μL) were dissolved in 2.5 mL of 100% ethanol (Sigma-Aldrich, St. Louis, MO, USA) to generate a 100 mM FFA stock solution. This solution was subsequently combined with 22.5 mL of 20% fatty acid-free BSA in phosphate-buffered saline (PBS) and incubated at 70 °C for 1 h, yielding a final concentration of 10 mM. Control solution containing 18% BSA was prepared by mixing 2.22 mL of 100% ethanol with 20 mL of 20% fatty acid-free BSA. All stock solutions were stored at −20 °C.

### 2.5. Cell Viability Assay

Cell viability was assessed using Cell Counting Kit-8 (CCK-8) (Dojindo, Kumamoto, Japan). HepG2 cells were seeded at 1 × 10^5^ cells/well in 96-well plates (SPL Life Science, Pocheon-si, Republic of Korea) and incubated overnight at 37 °C for attachment. Subsequently, cells were treated with various APE concentrations (0–400 μg/mL) or 0.125 mM FFA for 24 h. Cells received 10 μL of CCK-8 solution per well and were incubated for 2 h in a CO_2_ incubator. Absorbance was measured at 450 nm using a microplate reader (Bio-Tek Instruments, Winooski, VT, USA).

### 2.6. Oil-Red O Staining

To visualize lipid droplet accumulation in HepG2 cells, cells were seeded at 2.5 × 10^5^ cells/well in 12-well plates and incubated overnight. Cells were pre-treated with indicated APE concentrations for 2 h, then exposed to 0.125 mM FFA for 24 h. Following treatment, cells were fixed with 4% paraformaldehyde (PFA) at room temperature for 30 min, rinsed with 60% isopropanol, and stained with diluted Oil-Red O working solution for 10 min. Lipid droplets were visualized using an inverted microscope. For quantification, lipid droplets were extracted with isopropanol and absorbance was measured at 500 nm.

### 2.7. Triglyceride (TG) Assay

Intracellular TG levels were quantified using a TG quantification kit (MAK266, Sigma-Aldrich, St. Louis, MO, USA). HepG2 cells were seeded at 5 × 10^5^ cells/well in 6-well plates and incubated overnight. Following pre-treatment with indicated APE concentrations for 2 h and subsequent 0.125 mM FFA exposure for 24 h, cells were lysed in 200 μL of Triglyceride Reagent. Lysates were centrifuged at 10,000× *g* for 10 min at 4 °C, and supernatants were collected for TG quantification. Supernatants (10 μL) were mixed with 50 μL of Master Reaction Mix in each well and incubated at 37 °C for 30 min. Absorbance was measured at 570 nm using a microplate reader. TG concentrations were calculated using a standard curve generated with TG standards.

### 2.8. Animals and Experimental Protocols

**Figure 2:**
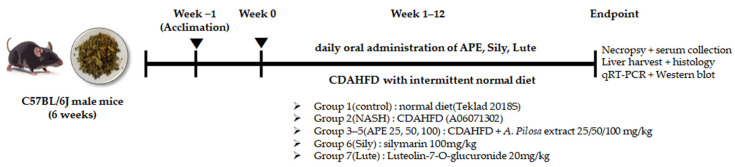
Experimental Scheme of In vivo Study. Overall experimental timeline showing acclimation, CDAHFD feeding period, daily oral administration of APE and reference compounds for 12 weeks, followed by endpoint tissue and serum analyses.

Male C57BL/6J mice (6 weeks old) were purchased from Joongang Experimental Animal Co. (Seoul, Republic of Korea) and housed in individually ventilated cages under controlled conditions (22–24 °C, 40–50% humidity) with a 12 h light/dark cycle. Following one week of acclimation, mice were randomly assigned to seven groups (*n* = 8 per group): Group 1 (Control): normal diet (Teklad 2018S, Envigo, Madison, WI, USA); Group 2 (NASH): CDAHFD (A06071302, Research Diets Inc., New Brunswick, NJ, USA); Groups 3–5 (APE 25, 50, and 100 mg/kg/day): CDAHFD with oral APE administration; Group 6 (Sily, 100 mg/kg/day): CDAHFD with oral silymarin administration; and Group 7 (Lute, 20 mg/kg/day): CDAHFD with oral luteolin-7-*O*-glucuronide administration (Figure 2). All treatments were administered for 12 weeks by oral gavage using a stainless-steel feeding needle, with all compounds freshly prepared in sterile water and delivered at 10 mL/kg body weight. To promote weight gain and induce chronic pathological changes in CDAHFD-fed groups, the diet was periodically replaced with a normal diet for one day per week. At the 12-week experimental endpoint, mice were anesthetized with isoflurane, and liver samples and blood from the portocaval vein were collected and stored at −75 °C for subsequent analysis. All animal procedures were approved by the Kyungpook National University Institutional Animal Care and Use Committee (IACUC) on 24 December 2021 (authorization no. KNU-2021-0224).

### 2.9. Measurement of Biochemical Markers

Blood samples were collected and centrifuged at 8000× *g* rpm for 30 min at 4 °C to isolate serum for liver function assessment. Serum aspartate transaminase (AST) and alanine transaminase (ALT) levels were analyzed using a DRI-CHEM NX500i biochemistry analyzer (Fujifilm, Tokyo, Japan).

### 2.10. Histological Analysis

Liver tissues were fixed in 10% neutral buffered formalin and embedded in paraffin. Paraffin-embedded liver sections (3 μm thickness) were processed for histological evaluation using hematoxylin and eosin (H&E) or Sirius Red staining according to standard protocols. For H&E staining, sections were deparaffinized in xylene, rehydrated through a graded series of alcohols, stained with hematoxylin for 4 min, counterstained with eosin for 3 min, dehydrated through graded alcohols, cleared in xylene, and mounted with Canada balsam (Sigma-Aldrich, St. Louis, MO, USA). For Sirius Red staining, deparaffinized and rehydrated sections were incubated with 0.1% Sirius Red (Direct Red 80; Sigma-Aldrich, St. Louis, MO, USA) in saturated picric acid at room temperature for 1 h, rinsed twice in acidified water, dehydrated through graded alcohols, cleared in xylene, and mounted with Canada balsam. All liver sections were examined using a Nikon ECLIPSE 80i microscope (Nikon Instruments Inc.,Tokyo, Japan). Fibrosis was quantified by measuring Sirius Red-stained areas in randomly selected 10× magnification fields using ImageJ software.

### 2.11. Immunohistochemical Staining (IHC)

Immunohistochemical analysis was performed to evaluate inflammation- and fibrosis-related protein expression in liver tissues. Paraffin-embedded sections were deparaffinized in xylene, rehydrated through graded ethanol, and rinsed with distilled water. Endogenous peroxidase activity was blocked by incubation with 3% hydrogen peroxide in methanol for 10 min. Antigen retrieval was performed by heating the sections in citrate buffer (pH 6.0; Dako, Agilent Technologies, Santa Clara, CA, USA) using a pressure cooker at 110 °C for 5 min. After cooling to room temperature, sections were processed with Histostain Plus Broad-Spectrum Kit (Invitrogen, Carlsbad, CA, USA) according to the manufacturer’s instructions. The sections were then incubated overnight at 4 °C with primary antibodies against F4/80, collagen I (COL I), and collagen V (COL V) (Abcam, Cambridge, UK). After rinsing with PBS, sections were incubated with a biotinylated secondary antibody for 30 min, followed by streptavidin–peroxidase conjugate for another 30 min. The immunoreactive signals were visualized using 3,3′-diaminobenzidine (DAB; Vector Laboratories, Burlingame, CA, USA) substrate for 5 min and counterstained with hematoxylin for 10 s. Following dehydration through graded ethanol and clearing in xylene, sections were mounted with Canada balsam. Images were captured using a Nikon ECLIPSE 80i microscope. F4/80-positive cells were quantified in five randomly selected fields per slide using ImageJ software (version 1.52, NIH, Bethesda, MD, USA).

### 2.12. Real-Time Polymerase Chain Reaction

Liver samples were homogenized using a FastPrep-24™ 5G Homogenizer (MP biomedicals, Solon, OH, USA). Total RNA was extracted from HepG2 cells and liver samples using TRIzol reagent (Life Technologies, Grand Island, NY, USA) following the manufacturer’s protocol. For reverse transcription, 2.5 μg of total RNA was converted to cDNA using SuperiorScript III RT Master Mix (Enzynomics, Daejeon, Republic of Korea) with incubation at 25 °C for 10 min, 42 °C for 60 min, and 85 °C for 5 min. Real-time PCR was performed using TOPreal™ qPCR 2X PreMIX (SYBR Green with low ROX) (Enzynomics, Republic of Korea) on a qTOWER^3^ G instrument (Analytik Jena, Jena, Germany). Thermal cycling conditions were: 95 °C for 10 min; 40 cycles of 95 °C for 10 s, 60 °C for 15 s, 72 °C for 30 s; and 72 °C for 7 min. Melting curve analysis verified primer specificity. Relative gene expression levels were calculated using the comparative Ct method (2^−ΔΔct^) with β-actin as the reference gene. Results are expressed as fold-change in gene expression relative to the control group. Primer sequences for target genes are listed in Table 1.

### 2.13. Western Blot

HepG2 cells and mouse liver samples were homogenized using a FastPrep-24™ 5G Homogenizer (MP biomedicals, Solon, OH, USA) in RIPA buffer (Millipore, Bedford, MA, USA) supplemented with protease inhibitors (Roche, Switzerland). Homogenates were centrifuged at 13,000× *g* rpm for 30 min at 4 °C, and supernatants were collected. Protein concentrations were determined by measuring absorbance using a microplate reader (Epoch™ Microplate Spectrophotometer, BioTek Instruments, Winooski, VT, USA). Equal protein amounts were loaded onto 7.5–12% SDS-PAGE gels and electrophoretically separated. Separated proteins were transferred onto PVDF membranes (Millipore, Bedford, MA, USA), blocked with 5% non-fat dry milk in Tris-buffered saline with 0.1% Tween-20 (TBST) at room temperature for 1 h, then incubated overnight at 4 °C with primary antibodies against specific proteins, including sterol regulatory element-binding protein 1c (SREBP-1c, MyBioSource, San Diego, CA, USA), CCAAT enhancer-binding protein alpha (C/EBPα, Santa Cruz, CA, USA), carnitine palmitoyltransferase 1A (CPT1a, Abcam, Cambridge, UK), sirtuin 1 (SIRT1, Abcam, Cambridge, UK), phospho-AMP-activated protein kinase (p-AMPKα, Cell Signaling, Beverly, MA, USA), AMPKα (Cell Signaling, Beverly, MA, USA), cyclooxygenase 2 (COX-2, Abcam, Cambridge, UK), alpha-smooth muscle actin (α-SMA, Sigma-Aldrich, St. Louis, MO, USA), nuclear factor erythroid 2-related factor 2 (Nrf2, Santa Cruz, CA, USA), heme oxygenase-1 (HO-1, Cell Signaling, Beverly, MA, USA), and β-actin (Cell Signaling, Beverly, MA, USA). Following thorough TBST washes, membranes were incubated with HRP-conjugated secondary antibodies at 1:1000 dilution in TBST with 5% skim milk at room temperature for 1 h. After washing, protein bands were visualized using SuperSignal West Pico PLUS Chemiluminescent Substrate (Thermo Fisher Scientific, Waltham, MA, USA) and captured using the C-DiGit Blot Scanner (Li-COR, Corp., Lincoln, NE, USA).

### 2.14. Statistical Analysis

Data are expressed as means ± standard deviation (SD). Statistical analysis was performed using one-way analysis of variance (ANOVA) followed by Dunnett’s test. *p* < 0.05 (*), *p* < 0.01 (**), *p* < 0.001 (***), *p* < 0.05 (#), *p* < 0.01 (##), and *p* < 0.001 (###) were considered statistically significant. Data were analyzed using Prism software Version 8.4.3 (GraphPad Prism Software, Inc., San Diego, CA, USA).

## 3. Results

### 3.1. Effects of APE and FFA on HepG2 Cell Viability

**Figure 3:**
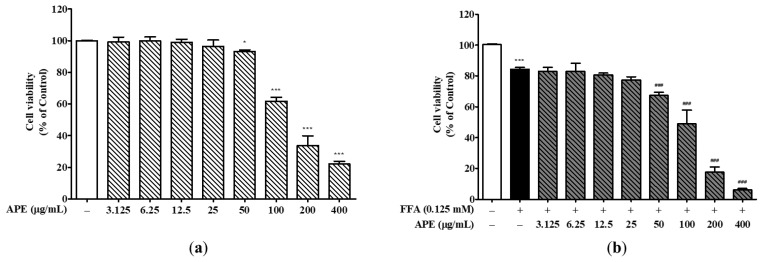
Effects of APE on HepG2 cell viability. (**a**) HepG2 cells were treated with various concentrations of APE (0, 3.125, 6.25, 12.5, 25, 50, 100, 200, and 400 μg/mL) for 24 h. (**b**) Cells were co-treated with 0.125 mM FFA and various concentrations of APE (0, 3.125, 6.25, 12.5, 25, 50, 100, 200, and 400 μg/mL) for 24 h. Cell viability was assessed using the CCK-8 assay. Data are presented as mean ± SD. * *p* < 0.05, *** *p* < 0.001 versus control group; ^###^ *p* < 0.001 versus FFA-treated group.

To investigate APE and FFA effects on cell viability, HepG2 cells were exposed to various APE concentrations either alone or combined with 0.125 mM FFA for 24 h. Results demonstrated that APE did not affect cell viability at concentrations up to 50 μg/mL. However, cell viability decreased notably by approximately 50% at 200 μg/mL (33.82 ± 5.22%) and further at 400 μg/mL (22.11 ± 1.46%) compared to the control, with both decreases being statistically significant (*p* < 0.001; Figure 3a). Treatment with 0.125 mM FFA alone reduced cell viability to 84.35% ± 0.93% of control levels, consistent with FFA-induced lipotoxicity (*p* < 0.001 vs. control). Co-treatment with APE and FFA further decreased cell viability in a concentration-dependent manner, with values of 49.00% ± 7.11% at 100 μg/mL, 17.66% ± 2.62% at 200 μg/mL, and 6.00% ± 0.81% at 400 μg/mL (all *p* < 0.001 vs. control; Figure 3b). Based on these results, cellular steatosis was induced in HepG2 cells using a fixed concentration of FFA, with APE co-treatment at concentrations ranging from 0 to 50 μg/mL.

### 3.2. Effects of APE on Intracellular Lipid Accumulation in HepG2 Cells

**Figure 4:**
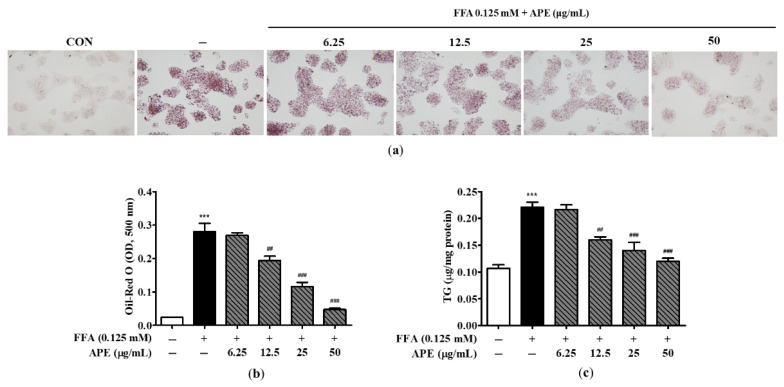
Effects of APE on intracellular lipid accumulation in HepG2 cells. HepG2 cells were pre-treated with the indicated concentrations of APE (0, 6.25, 12.5, 25, 50 μg/mL) for 2 h, followed by treatment with 0.125 mM FFA for 24 h. Cells were stained with Oil-Red O; (**a**) lipid droplet accumulation was visualized using a microscope (200× magnification). Red-stained areas indicate intracellular neutral lipid droplets stained by Oil-Red O. (**b**) Oil-Red O absorbance was quantified by measuring the optical density at 500 nm. (**c**) Intracellular TG contents were determined using a TG quantification kit. Data are presented as mean ± SD. *** *p* < 0.001 versus the control group; ^##^ *p* < 0.01, ^###^ *p* < 0.001 versus FFA-treated group.

To assess the effect of APE on lipid droplet accumulation, cells were pre-treated with the indicated concentrations of APE for 2 h and then treated 0.125 mM FFA for 24 h. Cells were subsequently stained with Oil-Red O, and lipid accumulation was quantified by measuring the absorbance at 500 nm. Morphological observations revealed a markedly greater accumulation of lipid droplets in HepG2 cells exposed to 0.125 mM FFA than in control cells, thereby confirming the successful induction of hepatic steatosis in this model. However, APE treatment effectively reduced lipid droplet accumulation, suggesting its potential as a therapeutic agent against hepatic steatosis (Figure 4a). Oil-Red O dye absorption in APE-treated cells decreased dose-dependently compared to FFA-treated cells (0.19 ± 0.018 nm at 12.5 μg/mL; *p* < 0.01, 0.11 ± 0.017 nm at 25 μg/mL and 0.04 ± 0.005 nm at 50 μg/mL; *p* < 0.001; Figure 4b). To evaluate the effect of APE on intracellular lipid accumulation, cellular TG levels were quantified using a TG quantification kit. Intracellular TG content was significantly elevated in FFA-exposed cells (0.22 ± 0.01 μg/mg; *p* < 0.001), whereas APE treatment markedly reduced TG levels at concentrations of 12.5–50 μg/mL (0.16 ± 0.008 μg/mg at 12.5 μg/mL; *p* < 0.01, 0.14 ± 0.021 μg/mg at 25 μg/mL; *p* < 0.001, 0.12 ± 0.008 μg/mg at 50 μg/mL; *p* < 0.001; Figure 4c). Collectively, these findings demonstrate that APE effectively mitigates intracellular lipid accumulation in HepG2 cells exposed to 0.125 mM FFA.

### 3.3. Effects of APE on Lipid Metabolism-Associated Gene and Protein Expression in FFA-Treated HepG2 Cells

**Figure 5:**
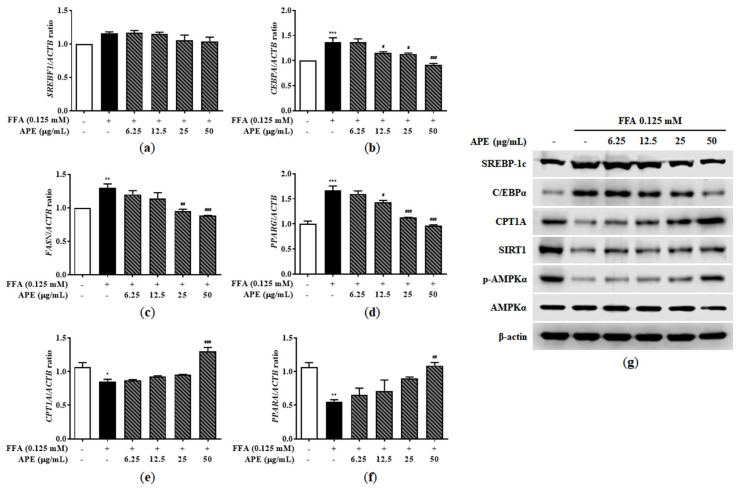
Effect of APE on lipid metabolism-related gene and protein expression in HepG2 cells. mRNA expression levels of (**a**) *SREBF1*, (**b**) *CEBPA*, (**c**) *FASN*, (**d**) *PPARG*, (**e**) *CPT1A*, and (**f**) *PPARA* were analyzed using quantitative real-time PCR and normalized to *ACTB*. (**g**) Protein expression levels of SREBP-1c, C/EBPα, CPT1A, SIRT1, phosphorylated-AMPKα, and AMPKα were determined using Western blot analysis. Data are presented as mean ± SD. * *p* < 0.05, ** *p* < 0.01, *** *p* < 0.001 versus the control group; ^#^ *p* < 0.05, ^##^ *p* < 0.01, ^###^ *p* < 0.001 versus the group treated with FFA alone.

The AMPK and SIRT1 signaling pathways play crucial roles in regulating metabolism and energy homeostasis, activating multiple regulators involved in fatty acid synthesis (including *SREBF1*, *CEBPA*, *FASN,* and *PPARG*) while simultaneously suppressing enzymes responsible for fatty acid oxidation (including *CPT1A* and *PPARA*). To identify underlying molecular mechanisms of lipid metabolism in HepG2 cells, we measured mRNA and protein expression levels of key regulators and enzymes involved in fatty acid synthesis and oxidation. In HepG2 cells, FFA exposure significantly upregulated mRNA expression of genes associated with fatty acid synthesis. *CEBPA* and *FASN* mRNA levels increased approximately 1.3-fold, and *PPARG* mRNA was elevated 1.66-fold in the FFA-treated group compared to controls (1.30 ± 0.08-fold for *FASN*, *p* < 0.01; 1.36 ± 0.12-fold for *CEBPA*, *p* < 0.001; 1.66 ± 0.12-fold for *PPARG*, *p* < 0.001). Although FFA treatment did not significantly alter *SREBF1* mRNA expression, APE treatment dose-dependently suppressed the upregulation of *SREBF1*, *CEBPA*, *FASN*, and *PPARG*. In the APE 50 μg/mL group, mRNA levels related to fatty acid synthesis were reduced to levels comparable with controls (0.91 ± 0.038-fold for *CEBPA*, 0.88 ± 0.008-fold for *FASN*, 0.96 ± 0.026-fold for *PPARG*; all *p* < 0.001 vs. FFA-treated group; Figure 5a–d). Fatty acid oxidation enzyme mRNA expression levels were significantly downregulated in FFA-treated cells compared to controls (0.85 ± 0.044-fold for *CPT1A*, *p* < 0.05; 0.55 ± 0.041-fold for *PPARA*, *p* < 0.01). However, APE treatment reversed this effect dose-dependently. High-concentration APE treatment (50 μg/mL) significantly increased *CPT1A* and *PPARA* mRNA expression by 1.3- and 1.1-fold, respectively, compared to controls (1.30 ± 0.081-fold for *CPT1A*, *p* < 0.001; 1.09 ± 0.064-fold for *PPARA*, *p* < 0.01; Figure 5e,f). Western blot analysis yielded similar results at the protein expression level. FFA treatment increased SREBP-1c and C/EBPα protein levels, while APE treatment, particularly at 50 μg/mL, markedly reduced this upregulation. Conversely, FFA exposure significantly reduced CPT1A, SIRT1, and phosphorylated AMPKα protein levels. APE treatment, however, significantly restored protein expression of phosphorylated AMPK, SIRT1, and fatty acid oxidation-related enzymes (Figure 5g). These findings suggest that APE alleviates hepatic lipid accumulation by inhibiting fatty acid synthesis and promoting fatty acid oxidation in FFA-induced steatosis.

### 3.4. Effects of APE on Body Weight and Liver Morphology in CDAHFD-Induced NASH Mice

**Figure 6:**
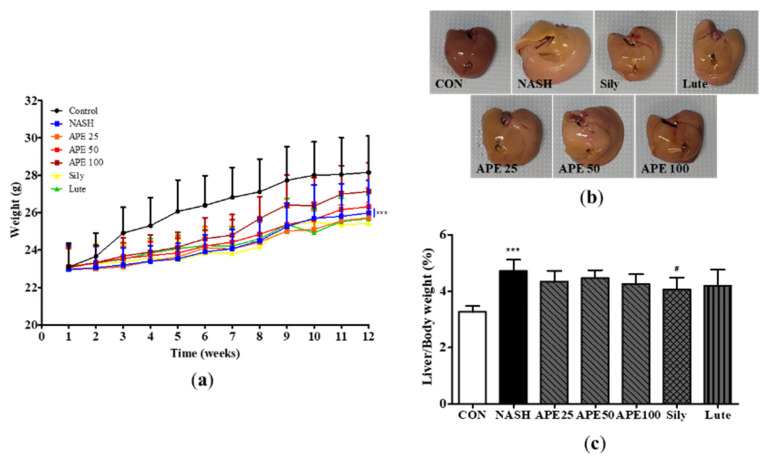
Effects of APE on mouse body weight and liver weight in a CDAHFD-induced NASH mouse model. (**a**) Body weight of mice in the CON (normal diet and vehicle), NASH (CDAHFD and vehicle), APE (CDAHFD and 25, 50, 100 mg/kg of APE), Sily (CDAHFD and 100 mg/kg of silymarin), and Lute (CDAHFD and 20 mg/kg of luteolin) groups over the course of 12 weeks. (**b**) Representative morphology of liver tissues. (**c**) Ratios of liver weight to body weight. Data are presented as mean ± SD. *** *p* < 0.001 versus the CON group; ^#^ *p* < 0.05 versus the NASH group.

To evaluate the effect of APE on CDAHFD-induced NASH, mice were provided CDAHFD supplemented with vehicle, APE, silymarin, or luteolin for 12 weeks. Changes in body weight and liver morphology were monitored. At the 12-week experimental endpoint, mean final body weights in the CDAHFD-induced NASH group and control group were 26.0 ± 1.6 g and 28.2 ± 1.8 g, respectively. The NASH group exhibited a significantly reduced final body weight compared to controls (*p* < 0.001). Administration of APE at 100 mg/kg slightly increased final body weight compared to the NASH group (27.1 ± 1.4 g; Figure 6a). Macroscopic liver appearance in the NASH group showed notable changes compared to controls, including color shift from reddish-brown to yellowish-brown and increased liver size, indicating steatohepatitis development induced by the CDAHFD. Remarkably, these morphological changes were substantially attenuated in the APE, silymarin, and luteolin treatment groups. Notably, APE administration at 100 mg/kg resulted in liver morphology closely resembling controls (Figure 6b). Additionally, liver-to-body weight ratio increased significantly in the NASH group (4.605 ± 0.451%) compared to controls (3.260 ± 0.203%; *p* < 0.001). However, liver-to-body weight ratios in the APE, silymarin, and luteolin groups were significantly lower than in the NASH group (4.058 ± 0.389% for silymarin; *p* < 0.05; Figure 6c).

### 3.5. Protective Effects of APE on Hepatic Steatosis and Liver Function in CDAHFD-Induced NASH Mice

**Figure 7:**
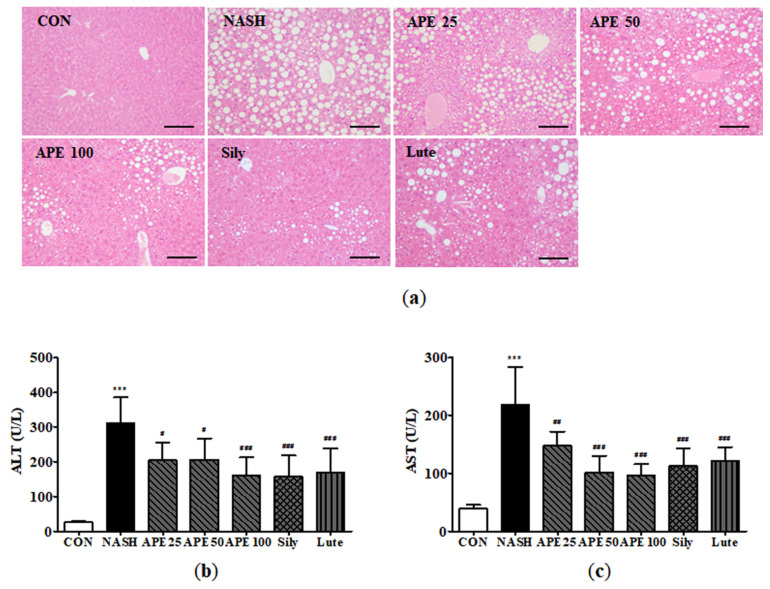
Effect of APE on hepatic steatosis and serum markers of liver injury in a CDAHFD-induced NASH mouse model. (**a**) Representative histological images stained with H&E (200× magnification). Serum levels of (**b**) ALT and (**c**) AST. Data are presented as mean ± SD. *** *p* < 0.001 versus the CON group, ^#^ *p* < 0.05, ^##^ *p* < 0.01, ^###^ *p* < 0.001 versus the NASH group.

To identify protective effects of APE against hepatic steatosis, we conducted histological examinations of liver tissues and analyzed serum ALT and AST concentrations, common markers for liver function and injury. Microscopic examination of H&E-stained liver sections from the NASH group revealed diffuse steatohepatitis, characterized by numerous lipid droplets, mild inflammation, and hepatocyte ballooning degeneration. In contrast, the APE, silymarin, and luteolin groups exhibited significant reduction in lipid droplets within hepatocytes and immune cells, indicating that APE treatment substantially inhibits hepatic steatosis and inflammation (Figure 7a).

Serum ALT and AST levels were significantly elevated in the CDAHFD-induced NASH group. Mean ALT level in the NASH group was 311.2 ± 66.7 U/L, compared to 26.0 ± 2.2 U/L in controls (*p* < 0.001). Similarly, the mean AST level in the NASH group was 219.0 ± 55.7 U/L, while controls showed 38.8 ± 6.4 U/L (*p* < 0.001). In treatment groups, APE (100 mg/kg), silymarin, and luteolin significantly reduced serum ALT and AST levels compared to the NASH group (*p* < 0.001). Among treatment groups, the APE 100 group showed a slightly more pronounced effect on both ALT and AST levels compared to other groups (ALT: 160.0 ± 49.1 U/L, *p* < 0.001; AST: 96.0 ± 18.7 U/L, *p* < 0.001; Figure 7b,c). These results suggest that APE exerts protective effects against hepatic steatosis and liver injury.

### 3.6. Effects of APE on Liver Inflammation in CDAHFD-Induced NASH Mice

**Figure 8:**
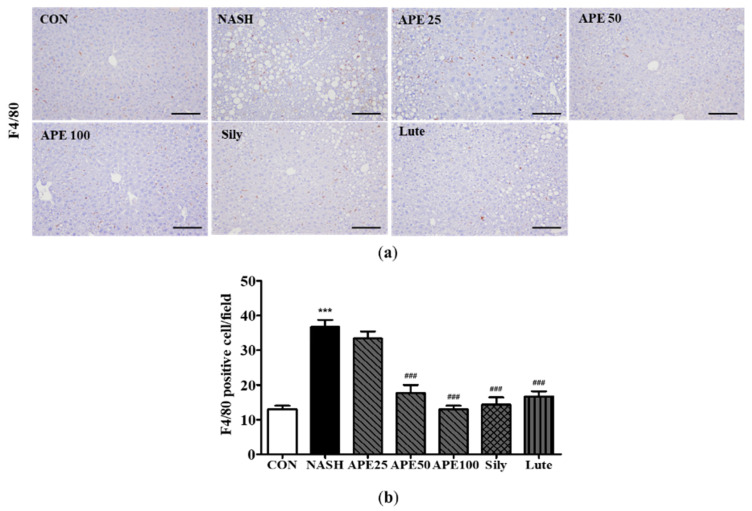
Effect of APE on liver immunohistochemistry for F4/80 positive cells in a CDAHFD-induced NASH mouse model. (**a**) Representative images of immunohistochemical staining (200× magnification). (**b**) Quantification of F4/80 positive cells. Data are presented as mean ± SD. *** *p* < 0.001 versus the CON group, ^###^ *p* < 0.001 versus the NASH group. Abbreviations: APE, *Agrimonia pilosa* extract; CDAHFD, choline-deficient, L-amino acid-defined, high-fat diet consisting of 60% kcal fat and 0.1% methionine; CON, control; NASH, non-alcoholic steatohepatitis; Sily, silymarin; Lute, luteolin-7-*O*-glucuronide.

To examine the effect of APE on liver inflammation in NASH, immunohistochemistry was performed to detect F4/80-positive liver macrophages. IHC results for F4/80 demonstrated that the control group contained a low number of F4/80-positive cells, whereas the NASH group exhibited substantially higher numbers, reflecting greater inflammatory activity (12.0 ± 1.41 cells in controls, 39.0 ± 3.52 cells in NASH; *p* < 0.001). Conversely, treatment with APE, silymarin, and luteolin resulted in reduced F4/80-positive cell counts compared to the NASH group (15.2 ± 2.78 cells for silymarin, 19.4 ± 3.61 cells for luteolin; *p* < 0.001). Remarkably, APE-treated groups exhibited dose-dependent decreases in F4/80-positive cells, with both APE 50 and APE 100 groups showing significant reductions (19.6 ± 3.20 cells for APE 50, 12.6 ± 1.01 cells for APE 100; *p* < 0.001; Figure 8a,b).

### 3.7. Effects of APE on Liver Fibrosis in CDAHFD-Induced NASH Mice

**Figure 9:**
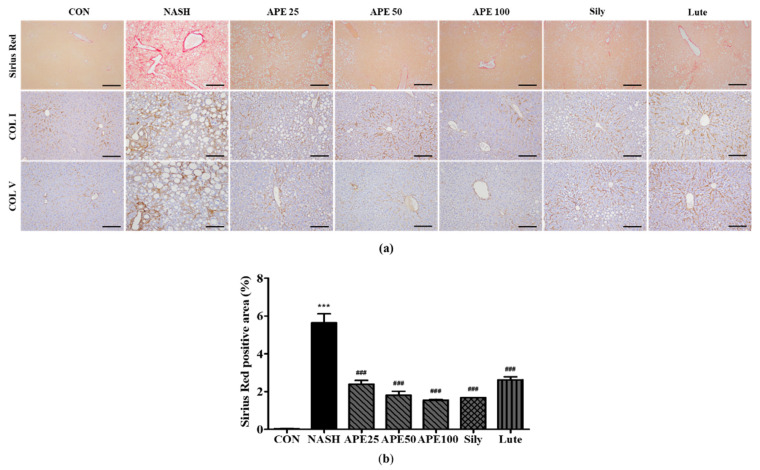
Effect of APE on liver histochemistry and immunohistochemistry for COL I and COL V in a CDAHFD-induced NASH mouse model. (**a**) Representative images of Sirius Red staining and immunohistochemical staining of COL I and COL V (200× magnification). (**b**) Quantification of the positive areas of Sirius Red staining. Data are presented as mean ± SD. *** *p* < 0.001 versus the CON group, ^###^ *p* < 0.001 versus the NASH group. Abbreviations: APE, *Agrimonia pilosa* extract; CDAHFD, choline-deficient, L-amino acid-defined, high-fat diet consisting of 60% kcal fat and 0.1% methionine; CON, control; NASH, non-alcoholic steatohepatitis; Sily, silymarin; Lute, luteolin-7-*O*-glucuronide; COL I, collagen I; COL V, collagen V.

To assess APE’s effects on liver fibrosis in NASH, Sirius Red staining and immunohistochemistry were performed to evaluate collagen deposition. Sirius Red staining revealed a significant increase in collagen deposition in the NASH group, indicating extensive liver fibrosis, compared to controls, which showed minimal collagen accumulation (0.03% ± 0.01% in controls vs. 5.63% ± 0.39% in NASH; *p* < 0.001). Treatment with APE, silymarin, and luteolin resulted in a substantial reduction in collagen deposition compared to the NASH group (1.69% ± 0.002% for silymarin, 2.63% ± 0.12% for luteolin; *p* < 0.001). APE treatment significantly reduced fibrotic staining dose-dependently across APE groups (2.39% ± 0.16% for APE 25, 1.81% ± 0.16% for APE 50, 1.54% ± 0.03% for APE 100; *p* < 0.001). IHC results for COL I and COL V followed similar patterns to Sirius Red staining. The NASH group exhibited marked increases in COL I and COL V expression, whereas controls showed minimal staining for both. Treatment with APE, silymarin, and luteolin led to notable reductions in COL I and COL V staining, with overall fibrosis level in the APE 100 group being comparable to controls (Figure 9a,b). These histological findings suggest that APE may be an effective therapeutic agent for reducing liver fibrosis in NASH.

### 3.8. Effects of APE on Lipid-Metabolism-Associated Gene and Protein Expression in CDAHFD-Induced NASH Mice

**Figure 10:**
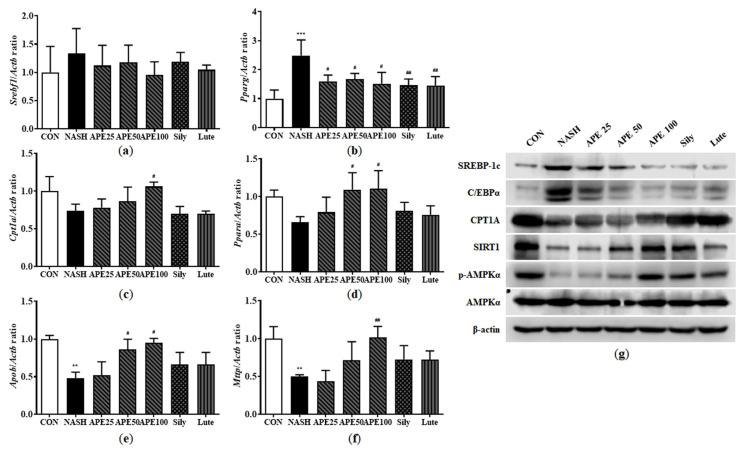
Effects of APE on the expression of lipid metabolism-related genes and proteins in a CDAHFD-induced NASH mouse model. mRNA expression levels of (**a**) *Srebf1*, (**b**) *Pparg*, (**c**) *Cpt1a*, (**d**) *Ppara*, (**e**) *Apob*, and (**f**) *Mttp* were analyzed via quantitative real-time PCR and normalized to *Actb*. (**g**) Protein expression levels of SREBP-1c, C/EBPα, CPT1A, SIRT1, phosphorylated-AMPKα, and AMPKα were determined via Western blot analysis. Data are presented as mean ± SD. ** *p* < 0.01, *** *p* < 0.001 versus the CON group, ^#^ *p* < 0.05, ^##^ *p* < 0.01 versus the NASH group.

To investigate molecular mechanisms underlying lipid metabolism in mouse liver, we analyzed mRNA and protein expression levels of enzymes involved in fatty acid synthesis, fatty acid oxidation, and very-low-density lipoprotein (VLDL) processing. Results showed that the NASH group exhibited elevated mRNA expression of *Srebf1* and *Pparg* by 1.33-fold and 2.48-fold, respectively, with *Pparg* showing a significant increase (*p* < 0.001 versus control). *Pparg* mRNA expression was significantly reduced in the APE, silymarin, and luteolin treatment groups compared to the NASH group in a dose-dependent manner. Specifically, mRNA levels in the APE treatment groups were 1.59 ± 0.17-fold for APE 25, 1.66 ± 0.16-fold for APE 50, and 1.51 ± 0.31-fold for APE 100, all *p* < 0.05 versus the NASH group. In contrast, the silymarin and luteolin groups showed 1.45 ± 0.17-fold and 1.45 ± 0.24-fold, respectively, (*p* < 0.01 versus the NASH group) (Figure 10a,b). Conversely, mRNA expression of *Cpt1a* and *Ppara* was reduced to 0.73 ± 0.07-fold and 0.65 ± 0.06-fold, respectively, in the NASH group. Treatment with APE at 100 mg/kg substantially increased *Cpt1a* and *Ppara* mRNA levels to 1.06 ± 0.04-fold and 1.10 ± 0.19-fold, respectively, (*p* < 0.05 vs. NASH) (Figure 10c,d). Additionally, mRNA expression of VLDL secretion-related genes, including apolipoprotein B (*Apob*) and microsomal triglyceride transfer protein (*Mttp*), was significantly reduced by approximately 0.5-fold in the NASH group (0.48 ± 0.06-fold for *Apob*, 0.50 ± 0.01-fold for *Mttp*; *p* < 0.01 vs. control). Nevertheless, APE treatment led to gradual increases in *Apob* and *Mttp* mRNA expressions. Particularly, mRNA expression of *Apo*b and *Mttp* in the APE 100 group were 0.94-fold and 1.01-fold, showing tendencies similar to controls (0.94 ± 0.04-fold for *Apob*; *p* < 0.05, 1.01 ± 0.24-fold for *Mttp*; *p* < 0.01; Figure 10e,f). Western blot analysis showed similar results for protein expression levels. The NASH group demonstrated increased SREBP-1c and C/EBPα protein levels compared to controls, and APE treatment gradually alleviated these protein levels. Conversely, CPT1A and SIRT1 protein levels and AMPKα phosphorylation were substantially decreased in the NASH group but were restored following APE, silymarin, and luteolin treatment. Moreover, APE treatment elevated SIRT1 protein concentrations and promoted AMPKα phosphorylation dose-dependently (Figure 10g). These results demonstrate that APE treatment regulates lipid metabolism by suppressing lipid synthesis and promoting lipolysis.

### 3.9. Effects of APE on Expression of Inflammatory Factors in CDAHFD-Induced NASH Mice

**Figure 11:**
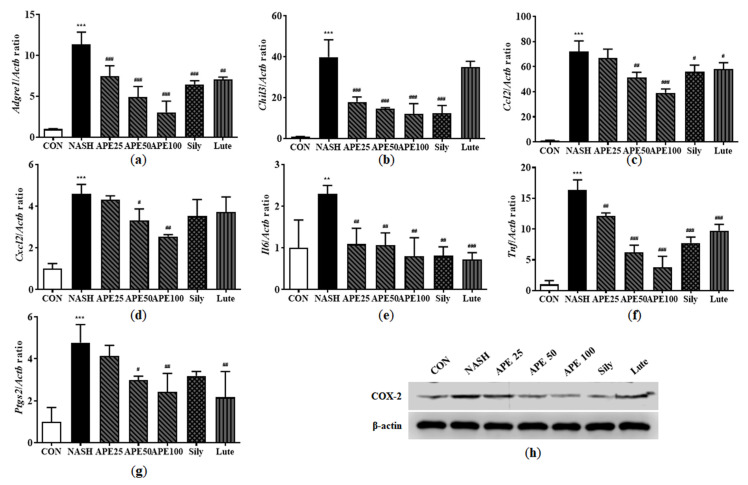
Effects of APE on the expression of inflammatory factors in a CDAHFD-induced NASH mouse model. mRNA expression levels of (**a**) *Adgre1*, (**b**) *Chil3*, (**c**) *Ccl2*, (**d**) *Cxcl2*, (**e**) *Il6*, (**f**) *Tnf*, and (**g**) *Ptgs2* were analyzed via quantitative real-time PCR and normalized to *Actb*. (**h**) The protein expression level of COX-2 was determined via Western blot analysis. Data are presented as mean ± SD. ** *p* < 0.01, *** *p* < 0.001 versus the CON group, ^#^ *p* < 0.05, ^##^ *p* < 0.01, ^###^ *p* < 0.001 versus the NASH group.

Inflammation is intricately involved in NASH progression, contributing to the transition from hepatic steatosis to steatohepatitis. To determine whether APE modulates hepatic immune responses, we assessed mRNA and protein expression levels of several inflammation-related markers, including the macrophage markers F4/80 and YM-1, the chemokines monocyte chemoattractant protein-1 (MCP-1) and macrophage inflammatory protein-2 (MIP-2), and the proinflammatory cytokines interleukin-6 (IL-6), tumor necrosis factor-α (TNF-α), and cyclooxygenase-2 (COX-2). The mRNA expression levels of the macrophage markers *Adgre1* (F4/80) and *Chil3* (YM-1) were significantly elevated in the NASH group, increasing by 11.34-fold and 39.91-fold, respectively (11.34 ± 1.24 for *Adgre1*; 39.91 ± 6.89 for *Chil3*; both *p* < 0.001 versus control). However, these markers were significantly reduced in APE-treated groups (3.04 ± 1.12-fold for *Adgre1*, 12.10 ± 4.09-fold for *Chil3* in the APE 100 group; *p* < 0.001; Figure 11a,b). Similarly, chemokines *Ccl2* (MCP-1) and *Cxcl2* (MIP-2) mRNA expression was dramatically higher in the NASH group, showing 72.11-fold and 4.60-fold increases compared to controls (72.11 ± 6.90-fold for *Ccl2*; 4.60 ± 0.36-fold for *Cxcl2*; *p* < 0.001). APE treatment significantly decreased these chemokines’ expression dose-dependently (39.19 ± 2.49-fold for *Ccl2*, *p* < 0.001; 2.55 ± 0.07-fold for *Cxcl2*, *p* < 0.01; Figure 11c,d). Proinflammatory cytokine mRNA expression, including *Il6*, *Tnf*, and *Ptgs2*, was also markedly increased in the NASH group compared to controls (2.29 ± 0.16-fold for *IL6*, *p* < 0.01; 16.33 ± 1.36-fold for *Tnf*; 4.77 ± 0.70-fold for *Ptgs2*; *p* < 0.001). APE treatment dose-dependently reduced the expression of these proinflammatory cytokines, with the APE 100 group showing the most significant decrease (0.79 ± 0.36-fold for *IL6*, *p* < 0.01; 3.76 ± 1.47-fold for *Tnf*, *p* < 0.001; 2.42 ± 0.71-fold for *Ptgs2*, *p* < 0.01; Figure 11e,g). Western blot analysis revealed similar results for COX-2 protein expression, with the APE 100 group demonstrating the greatest reduction, reaching levels comparable to controls (Figure 11h). These findings suggest that APE exerts significant anti-inflammatory effects in the liver by reducing chemokine and proinflammatory cytokine expression.

### 3.10. Effects of APE on Fibrosis-Related Protein Expression in CDAHFD-Induced NASH Mice

**Figure 12:**
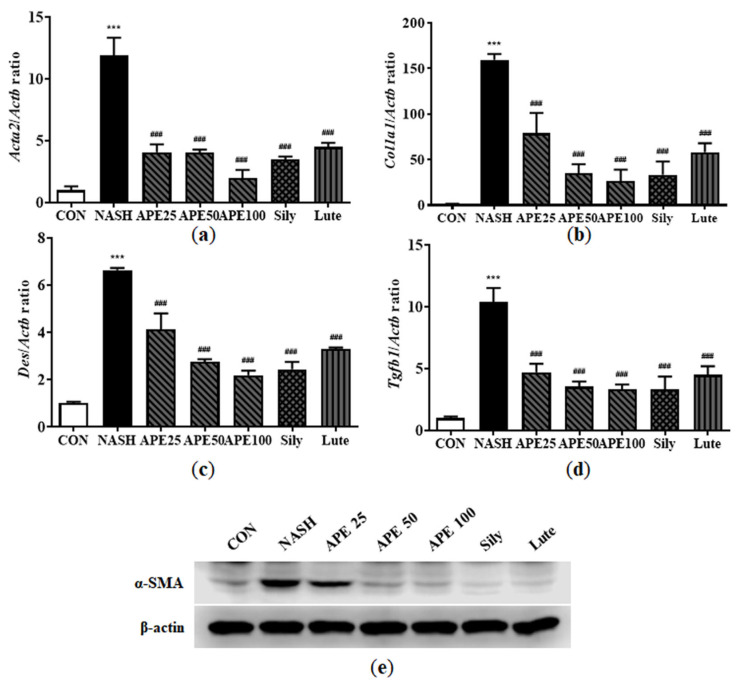
Effects of APE on the expression of fibrosis-related proteins in a CDAHFD-induced NASH mouse model. mRNA expression levels of (**a**) *Acta2*, (**b**) *Col1a1*, (**c**) *Des*, and (**d**) *Tgfb1* were analyzed via quantitative real-time PCR and normalized to *Actb*. (**e**) Protein expression level of α-SMA was determined via Western blot analysis. Data are presented as mean ± SD. *** *p* < 0.001 versus the CON group, ^###^ *p* < 0.001 versus the NASH group.

Hepatic fibrosis is caused by immune cell infiltration due to chronic inflammation. Inflammation triggers hepatic stellate cell (HSC) activation, leading to liver myofibroblast formation in NAFLD models. Activated HSC frequency expressing α-SMA and collagen type I alpha 1 chain (COL1A1) can be monitored to gauge liver fibrosis degree. However, α-SMA and COL1A1 positivity describes only a subpopulation of activated HSCs and myofibroblasts, so we also examined other myofibroblast markers, such as desmin and transforming growth factor beta 1 (TGF-β1). To investigate the hepatoprotective potential of APE against liver fibrosis, these fibrosis markers were examined via real-time PCR and Western blot analysis. Based on the mRNA levels of these markers, the NASH group exhibited significantly increased fibrogenic activity compared with controls (11.94 ± 1.15 for *Acta2*, 159.58 ± 5.20 for *Col1a1*, 6.64 ± 0.07 for *Des*, 10.42 ± 0.91 for *Tgfb1*; all *p* < 0.001 versus control). APE treatment dose-dependently attenuated the elevated mRNA expression levels of fibrosis markers, indicating a marked reduction in fibrotic potential. At the highest concentration, APE 100 significantly reduced expression levels (1.98 ± 0.54 for *Acta2*; 26.21 ± 10.43 for *Col1a1*; 2.19 ± 0.16 for *Des*; 3.31 ± 0.34 for *Tgfb1*; all *p* < 0.001 versus the NASH group; Figure 12a–d). Moreover, Western blot analysis of α-SMA revealed that its expression level was significantly higher in the NASH group than in controls. However, APE administration led to dose-dependent reduction in α-SMA protein expression level (Figure 12e). These findings demonstrate that APE can attenuate NASH-related fibrosis and HSC and myofibroblast activation in the liver.

### 3.11. Effects of APE on Oxidative Stress and Endoplasmic Reticulum Stress in CDAHFD-Induced NASH Mice

**Figure 13:**
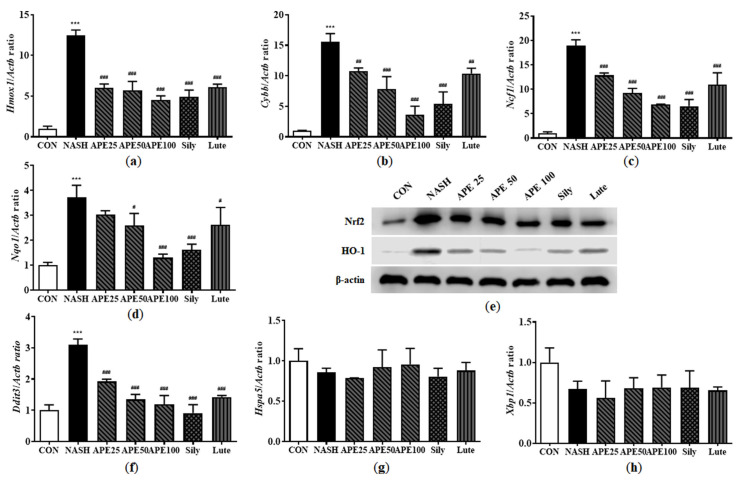
Effect of APE on the expression of oxidative stress and ER stress in a CDAHFD-induced NASH mouse model. mRNA expression levels of (**a**) *Hmox1*, (**b**) *Cybb*, (**c**) *Ncf1*, (**d**) *Nqo1*, (**f**) *Ddit3*, (**g**) *Hspa5*, and (**h**) *Xbp1* were analyzed via quantitative real-time PCR and normalized to *Actb*. (**e**) Protein expression levels of Nrf2 and HO-1 were determined via Western blot analysis. Data are presented as mean ± SD. *** *p* < 0.001 versus the CON group, ^#^ *p* < 0.05, ^##^ *p* < 0.01, ^###^ *p* < 0.001 versus the NASH group.

Progression from hepatic steatosis to NASH can be triggered by oxidative stress resulting from the overproduction of reactive oxygen species (ROS), as well as by endoplasmic reticulum (ER) stress. To assess whether APE can mitigate NASH-associated oxidative stress and ER stress, we performed quantitative PCR and Western blot analyses to measure the expression levels of stress markers involved in NASH progression. Results demonstrated that the NASH group had significantly increased mRNA expression levels of oxidative stress-related markers, including *Hmox1*, *Cybb*, *Ncf1*, and NAD(P)H dehydrogenase quinone 1 (*Nqo1*) (12.46 ± 0.53-fold for *Hmox1*, 15.55 ± 1.10-fold for *Cybb*, 18.88 ± 1.01-fold for *Ncf1*, 3.71 ± 0.39-fold for *Nqo1*; all *p* < 0.001 versus control). APE treatment dose-dependently reduced these elevated mRNA levels, with the most significant reductions observed in the APE 100 group (4.52 ± 0.42-fold for *Hmox1*, 3.66 ± 1.11-fold for *Cybb*, 6.91 ± 0.03-fold for *Ncf1*, 1.29 ± 0.11-fold for *Nqo1*; all *p* < 0.001 versus the NASH group; Figure 13a–d). Western blot analysis further showed that Nrf2 and HO-1 protein levels were significantly elevated in the NASH group compared to controls, and these were dose-dependently reduced by APE treatment (Figure 13e).

ER stress is another key factor in NASH pathogenesis and progression. While no significant changes were observed in apoptotic response regulators such as G protein-coupled receptor 78 (*Hspa5*) and X-box binding protein 1 (*Xbp1*) expression, growth arrest and DNA damage-inducible gene 153 (*Ddit3*) mRNA expression was significantly increased in the NASH group (3.10 ± 0.14; *p* < 0.001). However, APE, silymarin, and luteolin treatments resulted in significant reduction in *Ddit3* mRNA expression (1.19 ± 0.23 for APE 100, 0.90 ± 0.22 for silymarin, 1.42 ± 0.04 for luteolin; *p* < 0.001) compared to the NASH group (Figure 13f–h). Overall, these findings suggest that APE can inhibit NASH progression by mitigating oxidative stress and ER stress.

## 4. Discussion

NASH is a complex and progressive liver disease characterized by excessive hepatic lipid accumulation, chronic inflammation, oxidative and ER stress, and fibrotic remodeling. Owing to the multifactorial nature of its pathogenesis, therapeutic strategies targeting single signaling pathways have demonstrated limited clinical success. In this context, botanical extracts containing multiple bioactive constituents offer a promising alternative, as they can simultaneously modulate diverse pathological mechanisms while generally exhibiting favorable safety profiles.

In the present study, APE demonstrated pronounced hepatoprotective effects in both cellular and animal models of NASH. Initial in vitro screening identified luteolin-7-*O*-glucuronide as the most potent individual compound in protecting HepG2 cells against carbon tetrachloride-induced injury. Although luteolin derivatives have been reported to exert hepatoprotective effects in alcohol-induced liver injury and high-fat diet models [21,22], the role of luteolin-7-*O*-glucuronide in NASH has remained largely unexplored. Importantly, whole APE consistently exhibited superior efficacy compared with isolated luteolin-7-*O*-glucuronide, strongly suggesting synergistic interactions among multiple phytochemical constituents.

This finding is consistent with the emerging paradigm of “multi-component synergy” in phytomedicine [23], wherein the therapeutic efficacy of complex botanical extracts frequently exceeds that of isolated compounds. Similar observations have been reported for milk thistle extract compared with silybin alone [24] and for whole grape seed extract relative to isolated proanthocyanidins [25]. Collectively, these findings support the rationale for evaluating whole botanical preparations rather than focusing exclusively on single purified constituents.

To comprehensively evaluate the therapeutic potential of APE, we employed complementary experimental models, including FFA-induced steatosis in HepG2 cells and CDAHFD-induced NASH in mice. APE treatment significantly reduced intracellular lipid droplet formation and triglyceride accumulation in both systems. The magnitude of triglyceride reduction (approximately 45% in vitro and 38% in vivo) was comparable to that reported for berberine [26] and exceeded that reported for green tea catechins in analogous models [27]. Notably, the CDAHFD model used in this study more faithfully recapitulates key metabolic features of human NASH, including obesity, insulin resistance, and dyslipidemia, than the commonly used methionine- and choline-deficient diet model [28,29], thereby enhancing the translational relevance of the present findings.

Mechanistically, APE exerted potent anti-steatotic effects by suppressing lipogenic regulators such as SREBP-1c and C/EBPα while upregulating fatty acid oxidation genes, including CPT1a and PPARα. These coordinated changes were mediated through activation of the AMPK/SIRT1 signaling axis. AMPK activation has been widely recognized as a central mechanism underlying the hepatoprotective effects of various natural compounds, including resveratrol and berberine [30,31]. SIRT1 further promotes metabolic homeostasis through deacetylation of transcriptional regulators, thereby enhancing PPARα-driven fatty acid oxidation [32].

A notable aspect of the present study is the simultaneous activation of both AMPK and SIRT1 by APE. This dual-pathway modulation suggests a more comprehensive regulatory mechanism than that reported for many natural compounds that preferentially act through a single pathway [30,33]. In accordance with in vitro observations, APE administration to CDAHFD-fed mice significantly downregulated hepatic SREBP-1c expression while enhancing AMPK and PPARα signaling. The magnitude of SREBP-1c suppression achieved with APE was comparable to or exceeded that reported for silymarin and surpassed that reported for green tea catechins in similar experimental paradigms [17,34].

Beyond lipid metabolism, hepatic inflammation is a pivotal determinant of NASH progression. APE treatment markedly attenuated inflammatory responses, reducing hepatic expression of the macrophage marker F4/80, chemokine MCP-1, proinflammatory cytokines IL-6 and TNF-α, and the inflammatory mediator COX-2. The observed reductions in IL-6 and TNF-α were quantitatively similar to those reported for quercetin in MCD diet-induced NASH and comparable to those observed with omega-3 fatty acids in clinical studies [35,36]. Suppression of F4/80 and MCP-1 indicates effective inhibition of hepatic macrophage infiltration and activation, consistent with effects reported for anthocyanins and ginsenosides [37,38]. The pronounced suppression of COX-2 observed with APE may reflect synergistic targeting of multiple inflammatory signaling pathways, including NF-κB, MAPK, and JAK-STAT cascades [39,40,41].

Oxidative stress and ER stress are key contributors to NASH pathogenesis. APE administration significantly reduced hepatic expression of Nrf2, HO-1, and GADD153/CHOP in CDAHFD-fed mice. Because Nrf2 and HO-1 are typically induced as compensatory responses to oxidative stress, their downregulation likely reflects attenuation of the underlying oxidative burden rather than impairment of antioxidant defenses [42]. Similar normalization of stress-inducible antioxidant pathways has been reported in successfully treated NASH models using grape seed proanthocyanidins and astaxanthin [43,44]. The marked reduction in CHOP expression is particularly relevant, as elevated CHOP levels are closely associated with ER stress-induced hepatocyte apoptosis in NASH [45,46,47]. These findings suggest that APE alleviates ER stress indirectly by reducing upstream pathological triggers such as lipid accumulation, inflammation, and oxidative stress.

Progression from steatohepatitis to hepatic fibrosis represents a critical juncture determining long-term clinical outcomes. In the present study, APE treatment significantly reduced expression of fibrogenic markers, including α-SMA, COL1A1, desmin, and TGF-β1. The anti-fibrotic efficacy of APE was comparable to that reported for salvianolic acid B and glycyrrhizin and exceeded that reported for silymarin in experimental fibrosis models [48,49,50]. Reduction in desmin expression indicates effective inhibition of hepatic stellate cell activation, the central cellular event in hepatic fibrogenesis [51]. Concurrent suppression of TGF-β1, a master regulator of fibrogenic signaling [52], suggests that APE interferes with fibrosis progression at multiple levels by both limiting profibrogenic cytokine production and directly inhibiting stellate cell activation responses.

Collectively, these findings indicate that APE modulates multiple interconnected pathogenic mechanisms involved in NASH progression, including lipid metabolism dysregulation, inflammation, oxidative and ER stress, and fibrogenesis.

## 5. Conclusions

In conclusion, the present study demonstrates that APE exerts broad hepatoprotective effects in cellular and animal models of NASH. APE attenuated hepatic steatosis through AMPK/SIRT1-mediated regulation of lipid metabolism and effectively suppressed inflammation, oxidative stress, endoplasmic reticulum stress, and fibrotic responses. Notably, APE inhibited hepatic stellate cell activation and downregulated key profibrogenic mediators, indicating its capacity to interfere with fibrosis progression. The superior efficacy of whole APE compared with isolated luteolin-7-*O*-glucuronide supports the presence of synergistic interactions among multiple phytochemical constituents. Collectively, these findings suggest that APE represents a promising multi-target botanical candidate for further translational investigation in NASH.

## Figures and Tables

**Table 1:** Real-time PCR primers used in this study.

Genes	Forward Primer (5′–3′)	Reverse Primer (5′–3′)
Human		
*SREBF1*	GCGCCTTGACAGGTGAAGTC	GCCAGGGAAGTCACTGTCTTG
*CEBPA*	TGGACAAGAACAGCAACGAGTA	ATTGTCACTGGTCAGCTCCAG
*FASN*	CCCCTGATGAAGAAGGATCA	ACTCCACAGGTGGGAACAAG
*PPARG*	TGCAGGTGATCAAGAAGACG	AGTGCAACTGGAAGAAGGGA
*CPT1A*	CCTCCGTAGCTGACTCGGTA	GGAGTGACCGTGAACTGAAA
*PPARA*	ACGATTCGACTCAAGCTGGT	GTTGTGTGACATCCCGACAG
Mouse		
*S* *rebp* *1c*	GCTACCGGTCTTCTATCAATG	GCAAGAAGCGGATGTAGTC
*P* *parg*	AGGCCGAGAAGGAGAAGCTGTTG	TGGCCACCTCTTTGCTCTGCTC
*Cpt1a*	TCCACCCTGAGGCATCTATT	ATGACCTCCTGGCATTCTCC
*Ppara*	AGAGCCCCATCTGTCCTCTC	ACTGGTAGTCTGCAAAACCAAA
*ApoB*	GGACTGTCTGACTTCCATATTC	AAGACTTGCCACCCAAAG
*Mttp*	TCTCACAGTACCCGTTCTT	TCTTCTCCGAGAGACATATCC
*Adgre1* (F4/80)	CAACACTCTCGGAAGCTATTAT	GAATTCCTGGAGCACTCATC
*Chi3l3* (YM-1)	TCTGGTGAAGGAAATGCGTA	AATGATTCCTGCTCCTGTGG
*Ccl2* (MCP-1)	TGCTTCTGGGCCTGCTGTTC	ACCTGCTGCTGGTGATCCTCT
*Cxcl2* (MIP-2)	CACTGCGCCCAGACAGAAGT	GGGCTTCAGGGTCAAGGCAA
*I* *l* *6*	TAGTCCTTCCTACCCCAATTTCC	TTGGTCCTTAGCCACTCCTTC
*T* *nfa*	GCTGAGCTCAAACCCTGGTA	AGTACTTGGGCAGATTGACCT
*Ptgs2* (COX-2)	GCCTACTACAAGTGTTTCTTTTTGCA	CATTTTGTTTGATTGTTCACACCAT
Acta2 (α-SMA)	CTGACAGAGGCACCACTGAA	GAAGGAATAGCCACGCTCAG
*Col1a1*	TCCTCCAGGGATCCAACGA	GGCAGGCGGGAGGTCTT
*Des*	TACACCTGCGAGATTGATGC	ACATCCAAGGCCATCTTCAC
*Tgfb1* (TGF-β1)	TTGCTTCAGCTCCACAGAGA	TGGTTGTAGAGGGCAAGGAC
*Hmox1* (HO-1)	CACGCATATACCCGCTACCT	CCAGAGTGTTCATTCGAGCA
*Cybb* (NOX2)	TGGACGGCCCAACTGGGATA	CAAGGCTTCAGGGCCACACA
*Ncf1* (NOXA2)	CCGAGCAGGCCTTCACCAAA	TCCCACGAAGCTGCGTCAAG
*Nqo1* (NQO1)	TTCCCATTGCAGTGGTTTGG	CTGCTACGAGCACTCTCTCA
*Ddit3* (GADD153)	CTGGAAGCCTGGTATGAGGAT	CAGGGTCAAGAGTAGTGAAGGT
*Hspa5* (GRP-78)	CGAAGGGATCATCTGCTATTAC	CTTCATAGTCCTGCCCATTG
*Xbp1* (XBP-1)	TCCGCAGCACTCAGACTATG	ACAGGGTCCAACTTGTCCAG

Gene symbols are italicized and follow official HGNC (human) and MGI (mouse) nomenclature.

## Data Availability

The original contributions presented in the study are included in the article, further inquiries can be directed to the corresponding author.
